# Diet, Microbiota and Immune System in Type 1 Diabetes Development and Evolution

**DOI:** 10.3390/nu7115461

**Published:** 2015-11-06

**Authors:** María E. Mejía-León, Ana M. Calderón de la Barca

**Affiliations:** Department Nutrición y Metabolismo, Centro de Investigación en Alimentación y Desarrollo, A.C., Carr. La Victoria, Km. 0.6, Hermosillo, Sonora 83304, Mexico; esther.mejia83@gmail.com

**Keywords:** Type 1 diabetes, autoimmunity, diet, gut microbiota, dysbiosis, *Bacteroides*

## Abstract

Type 1 diabetes (T1D) is the second most frequent autoimmune disease in childhood. The long-term micro- and macro-vascular complications of diabetes are associated with the leading causes of disability and even mortality in young adults. Understanding the T1D etiology will allow the design of preventive strategies to avoid or delay the T1D onset and to help to maintain control after developing. T1D development involves genetic and environmental factors, such as birth delivery mode, use of antibiotics, and diet. Gut microbiota could be the link between environmental factors, the development of autoimmunity, and T1D. In this review, we will focus on the dietary factor and its relationship with the gut microbiota in the complex process involved in autoimmunity and T1D. The molecular mechanisms involved will also be addressed, and finally, evidence-based strategies for potential primary and secondary prevention of T1D will be discussed.

## 1. Introduction

Type 1 diabetes (T1D) is one of the two most frequent autoimmune disorders in childhood and adolescence. It is due to the cellular-mediated autoimmune destruction of pancreatic β-cells, which leads to an absolute insulin deficiency, disturbing glucose metabolism [1]. The T1D prevalence of 1:300 is increasing over the world, representing 5%–10% of all diabetes mellitus cases [2]. 

Long-term micro- and macrovascular complications of diabetes are the leading causes of mortality [3] and disability in young adults. Understanding T1D etiology will allow for the design of preventive strategies to avoid or delay T1D onset and help to keep it under control if developed.

Genetic predisposition is the main determinant involved in T1D development, with the human leucocyte antigen (HLA) DR3-DQ2 and DR4-DQ8 haplotypes as the most common variants involved, which are shared with other autoimmune diseases such as celiac disease [4]. Since pancreatic β-cell autoimmunity appears frequently in the first 6 years of life, and its progression towards T1D can occur in preschoolers or during puberty, the factors investigated as possible triggers are related to early life and the immune system maturation process [5,6]. In addition to genetics, other factors such as birth delivery mode, diet, infections, and the use of antibiotics have been associated with T1D development [7]. However, the causality and possible mechanisms by which these factors relate to T1D remain unclear.

During the last decade, advances in molecular techniques have allowed for the study of gut microbiota in animal models of T1D [8] and, more recently, in children with autoimmunity and T1D [9,10,11,12,13,14,15]. The gut microbiota could be the link between environmental factors and the development of autoimmunity and T1D. This has led to the proposal of a possible intestinal origin of T1D [9], and has placed the microbiota as the central factor for its study. 

In this review, we are focusing on the dietary element and its relationship with the gut microbiota in the complex process towards autoimmunity and the progression to T1D. The molecular mechanisms involved will also be addressed, and evidence-based strategies for potential primary and secondary prevention of T1D will be discussed.

## 2. Diet and the Shaping of the Gut Microbiota

The first gut microbiota composition is mostly acquired at birth. The delivery mode determines the type of microorganisms that will colonize the newborn gut. Thus, children born vaginally develop a microbiota composed by *Lactobacillus*, *Prevotella* or *Sneathia* spp. from the maternal vaginal tract. Meanwhile, in those infants born by caesarean section, the bacterial communities from the mothers’ or skin or the skin of participants in the surgical procedure, such as *Staphylococcus*, *Corynebacterium*, and *Propionibacterium* spp., will dominate [10].

After delivery, diet is one of the main modulators of infant gut microbiota. Diet acts in a direct way by providing the substrates and sources of bacterial contamination from breast and nipple skin in breastfeeding babies or due to the tools and preparation methods in bottle-fed babies with infant formulas. Diet also contributes indirectly in the regulation of intestinal and pancreatic physiology [11]. During early childhood, microbiota diversity rapidly increases and new strains are acquired. Breastfeeding increases the diversity of lactic acid bacteria, while infant formulas contribute to the acquisition of bacterial communities such as *Staphylococcus aureus, Clostridium difficile, Bacteroides* spp., and other pathogenic communities. The microbiota structure is very unstable until the age of 2–3 years and it responds to changes in the diet, such as the introduction of solid foods or diseases; in subsequent years, it resembles the adult composition [12,13]. 

Around the age of 7 years old, the most prevalent phyla are *Firmicutes* and *Bacteroidetes*, representing about 90% of microorganisms, while the remaining 10% consists of *Proteobacteria*, *Tericutes* and *Cyanobacteria*. Three enterotypes have been proposed for the world population, in accordance with with the clustering patterns seen in the variations in the levels of the dominant microbiota genera: *Bacteroides, Prevotella,* and *Ruminococcus* [14]. In adults, these enterotypes have been associated with long-term dietary patterns. Thus, the *Bacteroides* enterotype has been correlated with diets dominated by high levels of animal protein and saturated fats, as occurs in the western diet. On the other hand, the *Prevotella* enterotype is more prevalent in people with higher consumption of carbohydrates and simple sugars, as observed in agrarian and vegetarian societies [15]. These enterotypes appear to be stable in adults after 6 months despite changes in saturated fats and fiber in feeding patterns [16]. 

In the last 5 years, several studies have examined the microbiota of healthy school-age children from different regions around the world. In all cases, the age, dietary patterns, and geography/traditions were the main determinants explaining the differences in gut microbiota composition. For example, microbiota profiles rich in *Prevotella* have been described in children from Burkina Faso [17], Mexico [18], Indonesia [19], Thailand [19,20], Malawi [21], and Amerindians from the Venezuelan Amazon [21]. All of them have common diets with a low content of fat and animal protein, and a high content of starch, fiber, and plant polysaccharides. In contrast, in the same age group of the United States [21], Italy [17], China [19], Japan [19] and Taiwan [19], the present gut microbiota is dominated by *Bacteroides*. In these, the diet is westernized with a high content of animal protein and fat, and a low fiber content.

Recently, the enterotype hypothesis has been questioned and reformulated because, according to Knights *et al.* [22], the stability of microbiotal composition could arise because “people resemble themselves over time in general rather than because there are specific barriers to switching cluster types”. They demonstrated a temporal fluidity of enterotypes in a gradient form in which one individual can move across time. Hence, enterotypes can be unstable, continuous, and driven by sampling frame. Therefore, for a better understanding of the way that diet shapes the microbiota and in order to minimize bias, multiple sampling is recommended to avoid isolated “snapshots” of a dynamic process. It would also be useful to know the microbiotal composition throughout the entire gastrointestinal tract; however, this requires invasive techniques that are impractical in healthy people [23].

## 3. The Immunity-Diet-Microbiome Consortium: Towards T1D in Early Life

The modulation of the immune system by the microbiota begins even before birth. The intrauterine environment of the fetus during pregnancy is not completely sterile. There is evidence that the placenta of a term pregnancy has a non-pathogenic commensal microbiota in low-abundance, similar to the oral microbiome of non-pregnant women [24]. This suggests that from a very early stage, the fetus is exposed to bacterial antigens against which it has to develop tolerance.

The intestinal immune system begins to develop after 11 weeks of gestation. At 16 weeks, there are already functional B and T cells. However, the response to antigens remains blocked to protect the fetus from an overreaction of the immature immune system. This is possible because the amniotic fluid contains endotoxin-neutralizing histones and a lipopolysaccharide-binding protein that prevents the activation of the Toll-like receptor (TLR) pathway [25].

After birth, diet and microbiota are the decisive factors that guide the proper maturation of the immune system. Diet is a source of nutrients, but it is also the main route of entry for antigens to the organism [26]. At the same time, early colonizer microbiota produce stimuli that manage the differentiation of cells and tissues of the immune system [25]. At this stage, the infant immune system learns to distinguish the self from the non-self and to control the balance between regulatory and inflammatory responses in the host, due to the types of bacteria that form the gut microbiota [27]. 

To accomplish this, the immune system applies two adaptive anti-inflammatory strategies: first, the production of secretory IgA (sIgA) to prevent epithelial penetration and to control colonization over the surface towards the lumen. The second strategy is the development of oral tolerance, which helps to prevent hypersensitivity reactions against innocuous antigens that pass through the intestinal barrier [28].

A mutualistic relationship with the microbiota can occur because the gut epithelial cells express microbe-associated molecular pattern (MAMP) receptors, primarily TLR. The NF-κB pathway is activated by TLRs, producing a pro-inflammatory response. This results in the production of cytokines, chemokines, and antibacterial products, according to the type of TLRs that are activated and the microbiotal patterns that are being recognized. For example, the bacterial lipopolysaccharide (LPS) inhibits the interleukin-1 receptor-associated kinase (IRAK) M, a modulator of IRAK1, which is necessary for NF-κB activation. Similarly, the ubiquitination and degradation of IγB is inhibited by reactive oxygen species (ROS) which is induced by the microbiota and, peroxisome proliferator-activated receptor gamma (PPARγ), a product of the activation of Toll-like recepetor (TLR) 4 by LPS, diverts NF-κB from the cell core [29].

In this process initiated by bacterial recognition, there is also production of sIgA, differentiation of effector T helper (Th) 1, Th2, and Th17 cells, and the development of regulatory T cells (Treg). The differentiation of Tregs can be induced by commensal microbiota in the colon, such as Cluster IV and XIVa *Clostridia*, related to their short-chain fatty acid production, which stimulates the expression of Foxp3 in CD4+ T cells [27]. However, other bacterial communities can induce the production of inflammatory T cells. In this setting, the segmented filamentous bacteria can colonize the gut by getting in direct contact with the epithelium, facilitating their presentation by dendritic cells (DCs). This elicits a specific effector host response, characterized by a cascade of pro-inflammatory signals that culminate with the production of Th17 and Th1 cells, mediated by interleukin (IL)-1, IL-6 and IL-12, which can lead to autoimmunity [29]. Thereby, alterations in this process, such as dysbiosis or an inadequate introduction of foods during the first months of life, may increase susceptibility to and generate the development of autoimmune diseases, allergies and other disorders, locally in the gut or at a systemic level.

Besides this, the microorganisms of the microbiota can regulate the intestinal architecture, altering gut permeability. Epithelial cells are bound within each other by structural proteins such as zonulin, claudin, occluding, and actin [30]. For instance, enteropathogenic *E. coli* acts directly on the distribution of occludin and *Clostridium difficile* through its toxins A and B, can disorganize actin and dissociate the zonulin complex, increasing permeability by a paracellular route. Moreover, *Vibrio cholerae* produces the zonula occludens toxin, which is homologous and competes with zonulin causing loss of the tight junctions [31]. As a result, dietary antigens and microbiotal products can pass through the leaky gut and initiate the development of an autoimmune response in genetically predisposed individuals.

Dietary antigens associated with T1D depend on early feeding regimens, the age of introduction of foods, especially wheat, to the infant’s diet, and the current consumption of nutrients [26]. In contrast, breastfeeding has beneficial immunomodulatory effects in the newborn. Studies in mice have confirmed that passive-transferred sIgA prevents the translocation of bacteria in the intestine, promoting gut homeostasis, which protects against infection by pathogens [32]. In contrast, milk formula consumption has been historically associated with T1D. 

In Finnish children from the Diabetes Prediction and Prevention (DIPP) study [33] and in Americans from the Diabetes Autoimmunity Study in the Young (DAISY) study [34], it has been found that fat intake from bovine milk products as well as proteins from fresh milk presented an increase in the risk of advanced β-cell autoimmunity and subsequent progression to T1D. The presence of high titers of anti-β-casein at diagnosis of patients with T1D and with latent autoimmune diabetes of adults (LADA) has been shown [35]. The A1, A2, and B variants of the bovine β-casein contain the PGPIP (Pro-Gly-Pro-Ile-Pro) motif repeated several times in their sequence. This motif is also repeated in the glucose transporter GLUT2, present in the pancreas. Therefore, a possible explanation for pancreatic damage is a cross-reaction of the immune system initially directed against a dietary antigen. Meanwhile, in the sequence of human β-casein, proline is replaced with valine, avoiding the immunogenicity against the human protein [26].

In addition, it has been proposed that high-gluten diets could be one of the primary drivers for gut dysbiosis associated with the T1D development [36]. This is related with the timing and amounts of dietary gluten fed to infants. The progressive introduction of gluten-containing foods to the diet, in terms of quantity, between 3 and 7 months after birth, can decrease the risk of T1D-associated autoimmunity [37]. T1D children have an altered T-cell reactivity to wheat antigens in the gut and peripheral tissues [9]. Recently, we found that 96% and 20% of the studied T1D Mexican children presented high titers of IgG isotypes anti-gliadins and IgA anti-gliadins, respectively [38]. This was an expected finding since T1D shares its genetic HLA-associated risk with celiac disease. 

The effects of gluten on intestinal homeostasis are multiple. Gluten increases gut permeability, affecting the tight junctions, which is well described in celiac disease patients and recently in T1D. As a result, long gliadin peptides can move between the epithelial cells to the lamina propria. Then, the dendritic cells can detect them and migrate to other sites, such as the pancreatic lymph nodes, to activate autoreactive T cells [37]. In an *in vitro* study by Hamari *et al.* [39], it was found that pre-T1D children with multiple autoantibodies and those newly diagnosed with T1D presented a T-cell response against gliadin in a lower frequency and intensity than healthy controls and patients with long evolution T1D. This finding supports the idea of an aberrant immune response related to the development of T1D.

Considering all the results together, the interplay between diet, microbiota, and immune system could partly explain the origin of T1D in susceptible children. These mechanisms reflects the immune link between the pancreas and intestine, as they both develop from the embryonic endoderm [9]. 

## 4. The Diabetogenic Microbiome

Over the past decade, the first microbiotal studies in animal models suggested the novel possibility of T1D prevention in humans through gut microbiota modulation. Among them, the experiment by Brugman *et al.* [40], performed in Bio-Breeding diabetes prone (BB-DP) rats, proved that antibiotic treatment had an effect on the incidence of diabetes, and that the differences in gut bacterial composition were detectable before the rats developed disease. In another work by Wen *et al.* [8], the interaction between intestinal microbiota and the innate immune system was recognized as an epigenetic factor which can modify predisposition to T1D. In their study, specific-pathogen-free (SPF) non-obese diabetic (NOD) mice lacking myeloid differentiation primary response 88 (MyD88) did not develop T1D. The MyD88 protein is an adaptor for TLRs and other innate immune receptors that recognize microbial stimuli. Furthermore, they found that this effect was related to commensal microbiota, as germ-free MyD88-negative NOD mice developed diabetes. 

More recently, the antibiotic effects over the development of T1D has been further analyzed. It has been found that both the administration of broad spectrum antibiotics, which almost completely eliminate the commensal microbiota, and the use of selective antibiotics, which affect the microbiotal composition and limit certain bacterial groups, increase the incidence of T1D in NOD mice [41]. Moreover, fecal transplant from NOD to non-obese resistant (NOR) mice produced insulitis in the latter, and antibiotics accelerated the appearance of T1D in this model [42]. These studies suggest that antibiotics could potentiate the diabetogenic effects of the altered microbiome.

Research in this topic has strongly increased in the last five years. Taking advantage of the new high-throughput sequencing techniques and bioinformatics, studies in humans have been conducted, looking for the possibility of a diabetogenic microbiome. In Table 1, a comparison of the published studies to date is shown. It is remarkable that in all of these studies, the presence of dysbiosis has been related to the autoimmune process and its further progression to T1D. In Finnish patients, this imbalance has been associated with a decreased bacterial diversity after seroconversion, before the onset of hyperglycemia [43,44,45]. The development of β-cell autoimmunity may precede the appearance of hyperglycemia for over 15 years [5]. This represents a window of opportunity for possible microbiota-related therapies that could prevent or delay the development of T1D in autoimmune-presenting children. 

The pattern of relative abundance of gut bacteria is different among the conducted studies. However, regardless of ethnicity, age and geography, all studies have detected *Bacteroides* as the main genus leading to T1D-associated dysbiosis. An increased proportion of *Bacteroides* in White Americans [46] and Caucasian children [9,10,11,13] with beta-cell autoimmunity, as well at the onset of T1D of Mestizo children [18], has been reported. Furthermore, there is a directly proportional relationship between the number of T1D-associated autoantibodies and the abundance of *Bacteroides* [44,45]. Hence, the presence of a higher degree of dysbiosis could contribute to the fast progression towards T1D that occurs in children with multiple autoantibodies [5]. 

An interesting finding of Davis-Richardson *et al.* [47] was the identification of two specific species causing the increase in gut abundance of *Bacteroides*: *B. dorei and B. vulgatus,* with *B. dorei* significantly increased before seroconversion. Therefore, the authors proposed to use them as predictors of T1D-associated autoimmunity. However, no other studies exist that confirm the increased presence of these species in other populations or their power as a predictor tool for T1D. In addition, we found that T1D Mexican children [18] with more than 2 years of evolution, controlled with insulin, presented a lower *Bacteroides* abundance than children with T1D at onset. These patients also had a relatively increased abundance of *Prevotella*, approaching the microbiotal profile described for healthy children with the same age and population. 

Possibly, the full rebalancing of the proportion *Bacteroides* to *Prevotella* was not reached in our study [18] due to the diet of patients with T1D. The American Diabetes Association (ADA) [1] recommends an intensive insulin therapy scheme, using the carbohydrate counting method. This allows the patients to have a close to "normal" diet according to their customs. However, to achieve the goals of glycemic control, it tends to limit the dietary load and glycemic index, reducing carbohydrate intake and increasing fat consumption compared to healthy children. In the study by Virtanen *et al.* [48], 38 Finnish children with T1D were followed to analyze their diet. The proportion of energy from fats increased in these children from 26% at onset of disease to 30% two years later. The American Heart Association recommends limiting the intake of saturated fat to 7% of energy to prevent cardiovascular events [49]. In the same study [48], they found that most of the fat sources consumed by T1D children were of animal origin and the saturated fat consumption was around 11%, high above the recommended level. This suggests that the high fat diet may be maintaining the *Bacteroides* levels, limiting the full recovery of the microbiotal balance.

**Table 1:** Comparison of microbiota composition in humans with autoimmunity and T1D.

Country/Ethnicity	Diagnostic (*n*)	Age in Years	Microbiota Diversity in Autoimmunity/T1D	Microbiota Relative Abundance in Autoimmunity/T1D	Other Findings
Finland (DIPP Study)/Caucasians [43,50]	β-cell AI (4) HC (4)	0–2 0–3	Reduced	F/B ratio ↓ Increase in: ***Bacteroides*** genus, mainly *Bacteroides ovatus.* Decrease in: *Prevotella and Faecalibacterium*	Non-butyrate producers avoid optimal mucine synthesis in T1D-associated autoimmunity.
Spain/Caucasians [51]	T1D at onset (16) HC (16)	7.16 ± 0.72 7.48 ± 0.87	Similar to the control group (*p* > 0.05)	F/B ratio ↓ Increase in: *Clostridium, **Bacteroides** and Veionella.* Decrease in: *Lactobacillus, Bifidobacterium* and *Prevotella.*	Microbiota differences were associated with glycemic level.
Finland (FINDIA and TRIGR studies)/Caucasians [44]	β-cell AI (18) HC (18)	FINDIA/TRIGR: 5.1 ± 1/13.3 ± 1 FINDIA/TRIGR: 5.0 ± 2/12.8 ± 1	Reduced	F/B ratio ↓ Increase in: ***Bacteroides*** genus, *Clostridium perfringens.* Decrease in: *Bifidobacterium adolescentis* and *Bifidobacterium pseudocatenulatum*.	The abundance of lactate- and butyrate-producing bacteria was inversely related to the number of β cell autoantibodies.
Mexico/Mestizos [18]	T1D at onset (8) T1D ≥ 2 years evolution (13) HC (8)	12.3 ± 0.64 11 ± 1.04	Similar to the control group (*p* > 0.05)	Unaltered F/B ratio. Increase in: ***Bacteroides*** genus. Decrease in: *Prevotella, Acidaminococcus* and *Megamonas.*	The glycemic control in the T1D ≥ 2 years treated group partially normalizes the microbiotal profile towards *Prevotella*-dominant profile.
Finland (DIPP Study)/Caucasians [47]	High risk cohort (76): β-cell AI (29) T1D at onset (22) HC (47)	0–2	Reduced	F/B ratio ↓ Increase: in ***Bacteroides*** genus due to *Bacteroides dorei* and *Bacteroides vulgatus.*	*B.dorei* abundance peaked over 8 months prior to the appearance of the first islet auto antibody. It coincided with the introduction of solid foods.
Finland, Estonia (DIABIMMUNE Study)/Caucasians [45]	High risk cohort (33): β-cell AI (7) T1D at onset (4) HC (22)	0–3	Reduced	Increase in: *Blautia, Rikenellaceae, Ruminococcus gnavus* and *Streptococcus infantarius* in T1D cases at the time of alpha-diversity divergence.	Decreased community diversity occurs after seroconversion but before onset of T1D. T1D onset is preceded by increased inflammation-assoc. organisms and pathways.
USA/White Americans (TRIALNET Study) [46]	β-cell AI (21) T1D at onset (35) Seroneg. FDR (32) HC (23)	4–49 2–20 3–45 4–24	Similar to the control group (*p* > 0.05)	Increase in: ***Bacteroides.*** Decrease in: Prevotella * In seropositive subjects with multiple *versus* one autoantibody.	The microbiomes of β-cell AI and seroneg. FDRs clustered together but separate from those of T1D at onset and HC.

T1D: Type-1 Diabetes; β-cell AI: β-cell autoimmunity; F/B ratio: Firmicutes/Bacteroidetes ratio; HC: Healthy controls; FDR: First degree relatives. DIPP: Diabetes Prediction and Prevention; FINDIA: Finnish Dietary Intervention Trial for the Prevention of Type 1 Diabetes; TRIGR: Trial to Reduce Type 1 Diabetes in the Genetically at Risk; TRIALNET: Type 1 Diabetes TrialNet; * indicate that these findings (Increase in: *Bacteroides,* Decrease in: *Prevotella)* were only detected in seropositive subjects from the TRIALNET study with multiple versus one autoantibody and there were not considered the seronegative first degree relatives included in the original study design.

## 5. Microbiota: Molecular Mechanisms in T1D

To explain the pathways and the impact of T1D-associated dysbiosis in the metabolism, it is necessary to study the microbiota structural dynamics as an integral organ [52]. Understanding that the gut microbiota is an organ will make it possible to integrate its relationship with T1D as a key for designing new therapies to prevent and/or improve the T1D control. 

Dietary components provide different substrates that may result in several products during the fermentation processes. Changes in the microbiotal structure due to diet modifications are because some of the bacterial communities are “genetically better equipped” to metabolize those substrates. Moreover, the same substrate can be used in different pathways according to the type of bacteria that are colonizing the intestinal niche, or in relation to its abundance and available frequency [13]. An example of the former statement is lactate. This substrate can be transformed into butyrate or, in others, short chain fatty acids (SCFA) such as acetate, succinate, and propionate during its anaerobic bacterial fermentation in the gut, depending on the type of microbiota [50]. 

**Figure 1:**
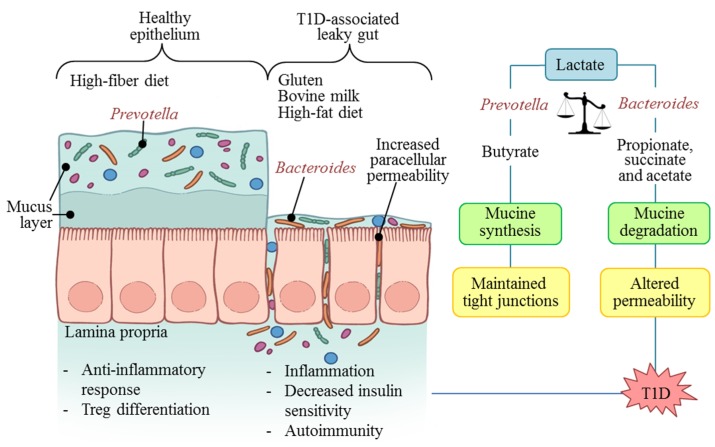
Diet and microbiota associated mechanisms in autoimmunity and type 1 diabetes (T1D) development.

The lactate model appears to be the strongest possible explanation for understanding the link between T1D and dysbiosis (Figure 1). According to this model, the presence of lactic acid- and butyrate-producing bacteria such as *Prevotella* and *Akkermansia* helps to maintain a healthy epithelium. This is because butyrate contributes to mucin synthesis and to the assembly of tight junctions [53]. These bacteria were common in the microbiota of healthy children around the world [9,10,11,12,15,50]. In contrast, when microorganisms such as *Bacteroides* and *Veillonella* are harbored in abundance, this substrate follows the pathway to succinate, acetate, and propionate. These products compromise mucin synthesis and increase paracellular permeability by altering the tight junctions [50]. 

In addition, butyrate may contribute to maintaining the anti-inflammatory response in the healthy gut by inhibiting the activation of NF-κB, signaling through G protein-coupled receptors, and leading to the modulation of antioxidant defense systems, nitric oxide production, and the expression of inflammatory cytokines [28,36]. High-fiber diets have been associated with a decreased risk of inflammatory immune-related diseases. However, it is unknown whether this effect is due to the butyrate itself or to the associated microbial profile [28]. Butyrate also enhances extra-thymic differentiation of Treg cells, while other SCFA, such as acetate, block this process [54]. Treg differentiation seems to be related to histone acetylation in the promoter of the Foxp3 locus, which is also regulated by butyrate [36]. This suggests that microbiota-derived products function as mediators in the communication between bacteria and the host immune system, leading to pro- or anti-inflammatory responses [54], and could be a factor involved in β-cell autoimmunity and T1D.

Systemic effects of intestinal butyrate in the regulatory immune response also occur at the pancreatic level. Butyrate has been associated with the expression of cathelicidin-related antimicrobial peptide (CRAMP) in the β-cells of NOD mice. This peptide has been shown to protect against the development of T1D by inducing a regulatory response and suppressing the inflammatory process in the pancreatic islets of prediabetic mice [55].

Free fatty acid receptor 2 (FFAR2) is one of the G protein-coupled receptors that can be activated by microbiota-derived butyrate. This receptor is involved in the insulin signaling regulation in adipose tissue and in the maintenance of energy homeostasis. Its activation promotes the secretion of GLP-1 in the intestine, the suppression of fat accumulation, and, therefore, increased sensitivity to insulin [56]. This is an interesting mechanism because even though the main problem in T1D is not insulin resistance, the diabetes accelerator theory puts it in context. This theory proposes that in T1D, body constitution, insulin resistance, and autoimmunity are three processes that accelerate the loss of beta-cells by apoptosis [57]. This theory arises when observing that children with autoimmunity that progress more quickly to T1D have greater insulin resistance than those non-progressors [58]. Thus, decreased production of butyrate in children with low levels of *Prevotella* in their gut microbiota could be contributing to T1D development. 

Kostic *et al.* [45], in the DIABIMMUNE study, followed children with high genetic risk for T1D from the first days following birth. Their results show associations between the gut bacterial communities and metabolic profile in young children, such as the levels of *Blautia* with long-chain triglycerides and *Ruminococcus* with short-chain triglycerides. Furthermore, these two microorganisms, which were abundant in children who progress to T1D, correlated positively with the presence of branched-chain amino acids such as valine, isoleucine, and leucine. Meanwhile, Oresic *et al.* [59] found that the dysregulation of lipid and amino acid metabolism precedes the appearance of glutamic acid decarboxylase and insulin autoantibodies in children who later developed T1D.

## 6. T1D Prevention and Control Possibilities

Children born with a genetic risk of T1D represent 30% of all births, but most of them do not develop the disease [60]. The risk increases considerably when β-cell autoimmunity appeared; according to the TEDDY study group [61], this can be attributed to the presence of two or more associated autoantibodies. With the appearance of autoantibodies, the risk for T1D increases up to 75% in the next 10 years and it is almost certain within 20 years [62,63]. Therefore, it is essential to implement effective prevention strategies at three levels of attention: primary prevention, before seroconversion; secondary prevention, when the β-cell autoimmunity is already present, trying to prevent or delay the T1D onset in predisposed children; and tertiary prevention, when the T1D is already present, to avoid complications [62].

### 6.1. Primary Prevention of T1D

Based on the evidence, the primary prevention of T1D should focus on modifiable perinatal factors, which theoretically could help to prevent not only T1D, but other autoimmune and allergic diseases in children. Thus, as the newborn initial microbiota is primarily obtained from the mother during birth and lactation, the possibility of considering maternal microbiome as the starting point has been suggested [52]. Thus, maintaining a healthy maternal microbiota, avoiding unnecessary cesareans, and the promotion of breastfeeding are important activities in which both mothers and health caregivers have to be educated.

Dominguez-Bello *et al.* [64] evaluated the possibility of modulating the gut microbiota from cesarean-born children. The newborns were exposed to a first natural inoculum, obtained from the *Lactobacillus*-dominated vaginas of their healthy mothers, in order to mimic the probable microbiota that they would have acquired if they were born vaginally. Preliminary results show that these babies have bacterial communities with an intermediate pattern between children born vaginally and those born by cesarean not receiving the inoculum. Moreover, regarding lactation, breastfeeding must be encouraged. It is also important that mothers have a varied and balanced diet while nursing. It was recently found that maternal consumption of red meats, meat products, and vegetable oils increases the risk of the baby developing autoimmunity and T1D in the following years [65]. In turn, in those who are exclusively milk formula-fed, supplementation with prebiotics and probiotics have proven to be effective in modifying the intestinal microbiota, resembling the profile of infants who are breastfed, stimulating the proper maturation and function of the immune system [11]. However, the effectiveness of these practices long-term has not yet been proven and it is not known whether they are sufficient to counteract the negative effects associated with the consumption of cow's milk proteins in early life.

Regarding bovine milk proteins contained in milk formulas, an option to prevent the development of autoimmunity in children at high risk might be weaning with a highly casein-hydrolyzed formula. To confirm this, the TRIGR study [66] is evaluating whether or not the use of hydrolyzed formula is safer than conventional milk formulas. The final endpoint of this study is in 2017, when participants turn 10 years old. This is a study with enough power to confirm or reject this theory and to provide the certainty required to direct preventive strategies. The possible benefits of highly hydrolyzed formulas in reducing the risk of autoimmunity with respect to conventional formulas are wide. Among them, they may avoid early exposure to intact bovine insulin, decrease gut permeability and thus the paracellular transit of foreign peptides, induce maturation of Tregs, decrease inflammation, and, with this, potentially contribute to maintaining the diversity of the intestinal microbiota.

### 6.2. Secondary and Tertiary Prevention of T1D

Once the autoimmune process has started, diet is the main known modifiable factor capable of changing the risk of developing T1D. The progression to T1D in children with β-cell autoimmunity is associated with the intake of total sugars, especially from sugar-sweetened beverages in those with a high-risk genotype [67]. The results from the latest clinical and experimental studies suggest that an effective measure in diabetes could be to target the treatment to the modulation of microbiota [68]. Considering the current information, dietary interventions should focus on having a greater impact on the metabolic function of the microbiota rather than on its composition [23]. Still, much remains to be understood about T1D etiology. In other diseases which also have an inflammatory gut background, the use of probiotics and prebiotics has been tested for their management. However, despite these strategies enabling the increase in the abundance of specific bacteria at the genus and species levels, changes in the overall composition of the microbiota are small and are kept only during the intervention period [69].

Other possible practices for prevention and/or treatment include fecal transplantation and the use of mucosa-protective drugs to manage leaky gut syndrome. Although fecal transplant is the only way to completely change microbiome, it is still unknown how unstable the new ecosystem could be, and therefore, the duration and efficacy of its effect in pre-T1D patients. It must also be considered that if fecal transplant would be performed without any dietary intervention, its effect, if significant, would be short-term. In T1D patients, fecal transplant accompanied by diet intervention could help achieve glucose control and recover microbiotal balance. Regarding mucosa-protective drugs, there are new drugs, such as gelatine tannate, that could be used as an intestinal barrier-modulating drug. According to the first trials, this drug may favor a physiological permeability, creating a bioprotective film by forming bonds with mucin, avoiding the aggressive penetration of bacteria, restoring functionality, and thus inducing an indirect anti-inflammatory effect [70].

Finally, in order to treat patients who do not respond adequately to conventional treatment, and investigating the possibility of remission and/or cure of the disease, other therapeutic strategies for T1D are pancreas transplant, pancreatic islet transplant , and, more recently, stem cell therapy [71]. Stem cell therapy seeks to take advantage of the regenerative capacity and immunomodulatory effects of the pluripotent cells. However, none of them have been effective in clinical practice alone in the long term. According to Chhabra and Brayman [72], safe stem cell strategies should be combined with other techniques, such as islet transplant, using the latest gene therapies and novel immunosuppressive and immunomodulatory drugs. In addition, the modulation of intestinal microbiota through fecal transplant and dietary intervention may help maintain the beneficial effects of the discussed techniques long term.

## 7. Conclusions 

The composition of the gut microbiota can be modulated by diet. This modulation can promote the proper maturation of the immune system, or, result in gut dysbiosis and aberrant immune responses that can lead to autoimmunity and T1D in predisposed children. Thus, dietary antigens and microbiota-derived products could be acting as triggers of T1D by promoting a pro-inflammatory and metabolic dysfunctional environment. The genus *Bacteroides* is the largest representative of T1D-associated dysbiosis. Among the possible strategies for prevention and treatment, fecal transplant accompanied by dietary intervention appears to be the most promising option for the prevention of T1D in children with autoimmunity.
